# Supported TiO_2_ in Ceramic Materials for the Photocatalytic Degradation of Contaminants of Emerging Concern in Liquid Effluents: A Review

**DOI:** 10.3390/molecules26175363

**Published:** 2021-09-03

**Authors:** Sadjo Danfá, Rui C. Martins, Margarida J. Quina, João Gomes

**Affiliations:** Chemical Engineering Processes and Forest Products Research Center (CIEPQPF), Department of Chemical Engineering, Faculty of Sciences and Technology, University of Coimbra, Rua Sílvio Lima, Polo II, 3030-790 Coimbra, Portugal; sadjodanfa@hotmail.com (S.D.); martins@eq.uc.pt (R.C.M.); guida@eq.uc.pt (M.J.Q.)

**Keywords:** photocatalytic processes, TiO_2_, supported catalysts, emerging contaminants, water reuse

## Abstract

The application of TiO_2_ as a slurry catalyst for the degradation of contaminants of emerging concern (CEC) in liquid effluents has some drawbacks due to the difficulties in the catalyst reutilization. Thus, sophisticated and expensive separation methods are required after the reaction step. Alternatively, several types of materials have been used to support powder catalysts, so that fixed or fluidized bed reactors may be used. In this context, the objective of this work is to systematize and analyze the results of research inherent to the application of ceramic materials as support of TiO_2_ in the photocatalytic CEC removal from liquid effluents. Firstly, an overview is given about the treatment processes able to degrade CEC. In particular, the photocatalysts supported in ceramic materials are analyzed, namely the immobilization techniques applied to support TiO_2_ in these materials. Finally, a critical review of the literature dedicated to photocatalysis with supported TiO_2_ is presented, where the performance of the catalyst is considered as well as the main drivers and barriers for implementing this process. A focal point in the future is to investigate the possibility of depurating effluents and promote water reuse in safe conditions, and the supported TiO_2_ in ceramic materials may play a role in this scope.

## 1. Introduction

Wastewater reclamation for multiple purposes is now seen as a central aspect of sustainable water resources management. This procedure has a strong impact both quantitatively, by relieving the pressure of decreasing volumes of freshwater required, and qualitatively, by reducing discharges of treated wastewater into sensitive areas [1,2]. However, the general criterion adopted to ensure water safety is that it should not contain substantial chemicals in toxic concentrations that may pose health and environmental threats [3]. In the current era of water scarcity, a consequence of drought and climate change as well as contamination, the effective treatment of wastewater becomes an important prerequisite for the economic growth and wellbeing of the population [1].

The contaminants of emerging concern (CEC) are compounds difficult to remove by the technologies implemented in conventional wastewater treatment plants (WWTP). These compounds are found in residual concentrations in liquid effluents and generally encompass pharmaceuticals and personal care products, pesticides, steroids hormones among others [4,5]. The presence of CEC in wastewater represents a major threat to both aquatic ecosystems and human health due to long-term exposure effect on non-target organisms as well as the promotion of bacterial resistance to these compounds (e.g., antibiotics). The occurrence of this class of contaminants in urban wastewater has been identified in various regions of the world. The most abundant belonged to the classes of drugs (e.g., antimicrobials, analgesics and anti-inflammatories, β-blockers, and lipid regulators) as well as personal care products [4]. Part of the pollutants in question can act as endocrine disruptors compounds (EDC), which can alter in some way the production, release, and activity of hormones in the body. Thus, several animal health problems can occur, such as breast or testicular cancer in humans. Besides, some studies have also shown the ability of these pollutants to cause changes in the reproduction of birds and fish [6]. The precautionary principle imposes that reusable water should not contain such compounds. As aforementioned, several investigations report that technologies currently implemented in the WWTP do not guarantee the efficient removal of this class of contaminants with concentrations in the orders of ng L^−1^ to µg L^−1^. In this view, promising processes have been tested, such as photocatalysis, ozonation, and biofiltration [7].

Heterogeneous photocatalysis is a low-cost and versatile advanced oxidation process (AOP), based on semiconductors (e.g., TiO_2_) with the ability to absorb ultraviolet/visible light. In the presence of water molecules, these catalysts can promote the generation of hydroxyl radicals (HO^▪^) and superoxide ions (O_2_^•−^), which are highly oxidizing leading to the degradation of pollutants [8,9].

According to Jabri and Feroz [10] TiO_2_ is a stable material and in general, the photocatalytic process does not need the addition of chemicals. Thus, this technology is of simple operation and economical. The most common photocatalyst is the commercial TiO_2_ powder (P-25). This material is usually used in suspension systems (slurry reactors). Thus, after the effluent treatment, methods of solid/liquid separation are required for its recovery. Here, technologies such as filtration, decantation, or centrifugation may be applied, which increases the complexity of the treatment operation and reduces its economic viability. Moreover, the application of powder catalyst makes difficult its reuse in continuous treatment processes.

According to the literature, the environmental application of supported photocatalytic semiconductors in the degradation of CEC in liquid effluents can bring short and long-term benefits in the use and sustainable management of water resources. In fact, the utilization of supported catalysts would allow the application of continuous operation treatment systems without the need for sophisticated solid/liquid separation units. This would overcome the major drawback associated to slurry photocatalytic systems. Clays, zeolites, and silica-based materials are common materials for incorporating TiO_2_, which demonstrates high chemical stability [11,12].

In this context, the objective of this work is to systematize and analyze the results of research inherent to the application of ceramic materials as support of TiO_2_ in the degradation of emerging contaminants in liquid effluents. To the best of our knowledge, there is a lack in the literature of a systematic overview regarding the use of supported catalysts in photocatalytic processes which highlights the novelty of the present work. Firstly, an overview of the presence of emerging contaminants in wastewater is given. Afterwards, the relevance of photocatalytic processes on the removal of these contaminants is highlighted and the theoretical foundations of this process are given. The disadvantages of using powder photocatalysts will be discussed and an overview of ceramic materials as TiO_2_ supports will be given. The application of such technology on the abatement of emerging contaminants and water disinfection is discussed with a special focus on water reuse.

## 2. Contaminants of Emerging Concern in Wastewater

CEC includes a diversity of compounds found in trace concentrations in industrial or municipal wastewater effluents. These pollutants comprise pharmaceuticals and personal care products (PPCP), polycyclic aromatic hydrocarbons, polychlorinated biphenyls, pesticides, microplastics, steroid hormones, and disinfection by-products, as well as the resulting processing products [4,13]. Indeed, the exponential growth of the population implies an increase in the use of PPCP, causing an increase in contamination of municipal effluents and other aquatic sources [14].

Gavrillescu et al. [15] highlighted that the production of new chemicals goes beyond current methods of safely monitoring and assessing inherent risks and technologies for their removal from wastewater. Many of these products are biologically active, of persistent nature, and with potential for bioaccumulation. Moreover, several molecules can react with other pollutants and generate new contaminants, representing a threat to ecosystems and human health [16]. Köck-Schulmeyer et al. [2] described the occurrence of pesticides in wastewater in Spain in noticeably high concentrations, in the order of 684 ng L^−1^ for diazinon. Moreover, they observed the environmental relevance of 22 pesticides for species of algae, daphnia, and fish. From the toxicity point of view, it was concluded, that the most problematic compounds were diazinon and diuron, followed by atrazine, simazine, malation, chlorotoluron, terbuthylazine, and isoproturon. Furthermore, Baalbaki et al. [17] emphasized the need to evaluate the capacity of WWTP to remove CEC due to discrepancies in removals reported in the literature that raised questions about the methods used to estimate pollutants degradation. In this study, the recently proposed “fractional approach” was used to explain the influence of hydrodynamics on WWTP and applied this method to estimate the removal of 23 CEC. As a result, all target CEC except triclosan were poorly removed in a primary clarifier, with efficiencies <30%. On the other hand, the activated sludge treatment in two different WWTP removed more than 70% of the CEC [17]. In fact, Alygizakis et al. [18] detected more than 279 CEC in treated wastewater after a membrane bioreactor. This highlights potential chemical and biological risks related to wastewater reuse practices when inadequate treatment processes are applied. In the same way, Qiu et al. [19] analyzed 20 antibiotics in water and sediment samples collected in the main rivers of Shenzhen, China. In this study, the identified CEC concentrations ranged from 36.5 to 1075.7 ng L^−1^ in 31 water samples and from 28.1 to 2728.8 ng g^−1^ in 31 sediment samples. In the Amazon estuary recognized as a Ramsar site, 11 PPCP were detected in water samples and 14 in sediments. Caffeine was the most frequent compound, reaching 13,798 ng L^−1^ [20]. Likewise, Lai et al. [21] tracked aquaculture systems and surrounding waters, concluding that six compounds occurred in relevant concentrations (mean values between brackets) namely, ibuprofen (788 ng L^−1^), lincomycin (624 ng L^−1^), flumequin (331 ng L^−1^), caffeine (276 ng L^−1^), ifosfamide (220 ng L^−1^), and cephalexin (172 ng L^−1^). Moreover, cross-contamination was found between these two environments. According to Zhang et al. [22], drug removal efficiency depends on WWTP technologies and the advancement of wastewater treatment technologies. This study highlighted that some conventionally recalcitrant chemicals can be effectively removed through WWTP. However, it is now known that the organic matter remaining in the effluents after conventional treatment may contain several recalcitrant xenobiotic organic compounds, including potential endocrine-disrupting compounds, antibiotics, among others [23].

It is important to note that CEC are not normally regulated in environmental legislation [24]. However, the reduction in surface water quality due to CEC contamination is an increasing concern and even more important when the objective is water reuse. In this sense, the development of technological processes that guarantee efficiency in the degradation of these compounds without the formation of dangerous intermediate products presents themselves as competitive solutions in the treatment of effluents. Therefore, the process of wastewater treatment with environmentally friendly methodologies and favorable cost-benefit has attracted the attention of several researchers [25]. The heterogeneous photocatalysis process has been highlighted due to its characteristics. This process is based on the potential of the highly reactive and non-selective hydroxyl radical in conducting oxidation processes for the complete mineralization of pollutants. Of course, other radicals and degradation pathways are involved in this technology. The production of these radicals is based on combinations of the catalyst (e.g., TiO_2_) and natural or artificial radiation. The results obtained in experiments with the photocatalytic treatment of liquid effluents showed quite satisfactory performance of TiO_2_ supported catalysts. Even though, the performance of this methodology must be evaluated in terms of its impact on the toxicity and disinfection capacity of the water [26]. Moreover, when dealing with heterogeneous catalytic reactions, the reusability performance of the catalysts is also an important parameter to evaluate the long-term efficiency of the photocatalytic material in practical applications [27]. As these cases are the main subject of analysis in this paper, their theoretical foundations will be presented in the following sections.

## 3. Heterogeneous Photocatalysis

Photocatalytic processes are interesting technologies for the CEC abatement. In this scope, TiO_2_ has been extensively studied for wastewater treatment due to its high photocatalytic activity, chemical stability, non-toxicity, and low cost [27]. Indeed, TiO_2_ reveals photocatalytic capabilities to absorb photons, and then promote the disintegration of different contaminants, by generating hydroxyl radicals among other moieties. The ability to degrade organic and inorganic pollutants comes from the redox environment generated by the photoactivation of TiO_2_, which is a semiconductor material [28].

**Figure 1:**
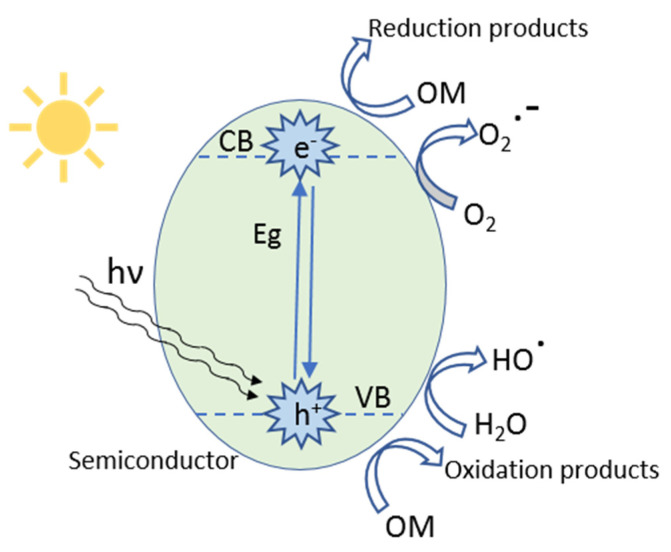
Scheme of the mechanism of heterogeneous photocatalysis (Image adapted, [30]).

A semiconductor is characterized by the possibility of excitation of electrons from valence bands (VB) to the conduction bands (CB), originating the appearance of holes in the valence band, and occupied states in the conduction band (Figure 1). Thus, there are negative mobile charges and consequently electrical transport in the CB and VB [29].

Equations (1)–(8) describe the reactions that may occur during the heterogeneous photocatalysis process of organic molecules (OM) using TiO_2_ as a catalyst, which generates hydroxyl and superoxide radicals. The VB promotes oxidation reactions for degradation of OM and CB aids reduction reactions. The presence of dissolved O_2_ is important as it can hinder the recombination of e^−^_(CB)_ and h^+^_(VB)_, maintaining the electroneutrality of the catalyst.

TiO_2_ + h → e^−^ (CB) + h + (VB) (1)

h^+^_(VB)_ + H_2_O → HO^▪^ + H^+^ (2)

h^+^_(VB)_ + OH^−^ → HO^▪^ (3)

h^+^_(VB)_ + OM → Oxidation products (4)

e^−^_(CB)_ + O_2_ → O_2_^▪−^ (5)

O_2_^▪−^ + H^+^ → HO_2_^▪^ (6)

OM + e^−^_(CB)_ → Reduction products (7)

Radicals (HO^▪^, HO_2_^▪^) + OM → Reduction products (8)

TiO_2_ conduction and valence bands are separated by different energy bandgaps dependent upon the crystalline phase. The energy bandgap (Eg) goes from 3.2 eV for anatase and 3.0 eV for the rutile phase, corresponding to the absorption of radiation at 387.5 and 400 nm in the ultraviolet region, respectively [9,31]. Silva [32] showed that the process of photocatalysis is heterogeneous, with some limitations that need to be addressed, such as the rapid recombination of photoinduced loads and low efficiency under sunlight. Therefore, several elements such as noble metals and transition metals, as well as non-metals and metalloids (i.e., graphene, carbon nanotube, and carbon quantum dots) are doped in the photocatalyst to improve the performance of photodegradation [33].

The effectiveness of the photocatalytic reaction depends on the type of TiO_2_ used, with a mixture of rutile (30%) and anatase (70%) showing to be highly reactive [33]. The commercial catalyst P25 is the most used, and its crystalline composition contains about 78% anatase and 14% rutile, the remaining 8% corresponds to the amorphous phase [34]. The applicability of TiO_2_ at lab-scale already proved high potential for wastewater treatment. However, its application at the industrial scale is far from proving this success rate. In part, the application of TiO_2_ in the wastewater treatment industry as a powder in nanoparticles needs a separation step after photocatalytic oxidation reaction which can represent relevant costs of installation and operation. Therefore, the immobilization of such nanoparticles in suitable supports as ceramic materials can eliminate the need for this separation stage. Considering this, the application of supported TiO_2_ instead of powder can be a key aspect for this material to be widely used in wastewater treatment at an industrial scale.

### 3.1. Radiation Source

The radiation source is an important point while designing a photocatalytic treatment process for depurating liquid effluents. The average solar energy incident on the earth’s surface is about 240 W m^−2^, part of which (about 5%) corresponds to UV-A and UV-B radiation with wavelengths from 290 to 400 nm [35]. Thus, UV irradiation provided by the sun is strong enough to cause a photocatalytic reaction in TiO_2_ (Equations (1)–(8)) [36]. Usually, ultraviolet radiation is subdivided into four regions: UV-A, UV-B, UV-C, and UV-Vacuum. UV-A and UV-C are the most widely used in environmental applications for water or wastewater treatment [37].

#### 3.1.1. UV Radiation

Ultraviolet (UV) radiation is generally defined as radiation with a wavelength between 100 and 400 nm. UV-C is more energetic and can be absorbed by water according to the following reaction:

(9) $$H_{2}O+h\nu (\lambda <190\;nm)\to H^{￭}+{\mathrm{HO}}^{￭}$$

Equation (9) indicates that the hydroxyl radical can be produced in the presence of this type of radiation without a catalyst. Lamps of different radiation wavelengths, especially of ultraviolet nature (UV-C, UV-B, UV-A), have been applied in the photocatalytic process [37]. This different radiation wavelength has an impact in terms of energetic consumption as well as on the process efficiency. The use of UV lamps allowed combinations with different systems to generate hydroxyl radicals, some examples are the combinations UV/H_2_O_2_, UV/O_3_, UV/TiO_2_. These processes allow obtaining good results in the secondary treatment of effluents enhancing the action capacities of the UV radiation [37].

Reactors with lamps that emit UV-A, UV-B, and UV-C radiation have been applied for the degradation of different classes of pollutants in aqueous solutions by immobilization of TiO_2_ in different supports [38,39]. The application of UV-C radiation has the handicap of high energetic consumption [40]. Alternatively, commercially available UV-A LED has proven to be very stable and with high photonic efficiency [41,42,43]. Contrarily, some studies have highlighted the limitations of UV-A due to low photonic efficiency in photocatalytic degradation of organic pharmaceutical pollutants in wastewater, such as for example when applying a diode lamp (TiO_2_/UV-A/LED) for the degradation of acetaminophen [44]. Other researchers tested the coupled effect of UV-A and UV-C (LED) on microbiological and chemical pollution of urban wastewater [44]. The authors concluded that LED (UV-A/UV-C) coupling provided a microbial reduction in wastewater more efficiently than when LED (UV-A or UV-C) was used alone and oxidizes up to 37% of creatinine and phenol, a result comparable to that normally obtained when a photocatalyst such as TiO_2_ is applied.

To reduce energy costs, the application of higher wavelength radiation may be a solution. However, for this, it is important to use a suitable semiconductor so that the production of reactive oxidative species may be possible [29]. For example, Jallouli et al. [45] studied the degradation of naproxen (NPX) by direct photolysis in an aqueous solution as well as by photocatalysis through the usage of TiO_2_ and UV-C radiation. The removal of NPX by photolysis was 83% after 3 h, with an 11% reduction in chemical oxygen demand (COD), while the photocatalytic oxidation led to higher removal of NPX (98%) and COD (25%) after the same time.

The results obtained from the kinetic analysis showed that UV-C/TiO_2_ oxidation is a promising process for treating CEC in water [46]. The experiments indicated that photocatalytic degradation of furfural in aqueous solution is efficient using granular activated carbon (GAC), with TiO_2_ and UV-C. The combination UV-C/TiO_2_/GAC was feasible and under optimal operational conditions, the furfural removal was 95% [47]. Other studies have explored the possibility of using UV-A and UV-C combined with oxidants (O_3_, H_2_O_2_) to investigate the degradation of sulfolane in water [48]. The authors found that UV-A photons are poorly absorbed, while UV-C is effectively absorbed by both oxidants, thus generating more HO^▪^ (and/or other reactive oxygen species). Regarding TiO_2_ activation, UV-B is more efficient than UV-A [8,49]. Slow kinetics with UV-A radiation has been observed in several simulated effluent treatment processes using TiO_2_ as a photocatalyst. This partly reinforces the importance of doping it with appropriate materials to enhance its photoactivity at low-energy radiation sources [50]. For example, Durán-Álvarez et al. [51]) found good results during the photocatalytic degradation of ciprofloxacin using mono (Au, Ag, and Cu) and bi- (Au-Ag and Au-Cu) metal nanoparticles supported on TiO_2_ under UV-C. However, using UV-C radiation with bimetallic materials a remarkable increase in photodegradation of the antibiotic compared to pristine TiO_2_, which required 90 min for the complete degradation of ciprofloxacin [51]. Other studies evaluated the photocatalytic degradation of simulated wastewater using four sources of radiation, UV-A, UV-B, UV-C, and a solar lamp [52]. The UV-C radiation proved to be the most effective, producing 100% Methylene Blue (MB) degradation within 14 min [52]. The degradation of azithromycin by TiO_2_ including radiation sources UV-A and UV-C range was also evaluated [53]. The most effective degradation process has been achieved with the UV-C radiation in matrices at pH 10. In other studies, a photocatalytic paint containing TiO_2_ was applied to substrates such as plastic Petri dishes and glass bottles for degradation of MB under the action of sunlight and UV-B [54]. Under UV-B light, it takes 120 min to degrade about 80% of 6 ppm MB while under sunlight it took 60 min to degrade about 90% of the same MB solution. Moreover, TiO_2_ photocatalyst can also be applied for efficient inactivation of *Escherichia coli* [55]. In this case, the results showed that the combination of TiO_2_ and UV-B light leads to the disruption of the outer membrane which causes the effective inactivation of *E. coli* bacteria.

It is important to note that some studies have demonstrated that photocatalytic processes are not characterized by a linear evolution of efficiency-irradiation. In view of this, to optimize the design of the processes it is important to adjust the flow of photons and sources of irradiation to the catalyst substrate and type of pollutant [56]. Globally, in most cases, high efficiency is observed by using UV radiation in the presence of TiO_2_ catalysts for the abatement of CEC from water [51]. Martins et al. [57] verified the effect of using different radiation sources and the UV-A radiation presented higher efficiency in CEC abatement comparing with visible radiation. However, it is important to consider the economic impact of this type of radiation on wastewater treatment. In this way, other radiation sources with lower operation costs must be considered.

#### 3.1.2. Visible Radiation

Aiming industrial processes applications, the photocatalyst should be active under the visible light spectrum since this is the largest fraction of sunlight radiation [56,58]. The mechanism for photocatalytic activity of TiO_2_ under visible light radiation depends on the electronic properties of TiO_2_ (crystalline phases, co-doped or multidoped, crystallite size, etc.) and the molecular structure of the organic compounds to be degraded [59,60]. To improve light absorption by catalysts in the visible light region, one of the alternatives is doping it with metallic (such as Cu, Ni, Fe), and non-metallic (N, S, B) ions for reduction of the catalyst bandgap [29,61]. The Cu-doped TiO_2_ enhanced the photocatalytic oxidation of phenol compared to the bare TiO_2_ under visible light radiation [61]. Wang et al. [62] proved that the reduced graphene oxide/TiO_2_ doped with rare metals can degraded phenol under a weak visible light radiation. Moreover, some noble metals, due to their plasmonic resonance, can also efficiently absorb visible light [63,64,65]. Vega et al. [66] using doping of TiO_2_-Ni for the degradation of *p*-Arsanylic Acid (*p*-ASA, 10 mg L^−1^) in aqueous solutions under visible radiation. The authors found that TiO_2_ -Ni showed higher photocatalytic activity than pure TiO_2_, promoting the degradation of 76% of *p*-ASA, while 60% of the degradation was achieved with non-doped TiO_2_. Some authors verified the successful deposition of Pd and Pt atoms on the surface of TiO_2_ that allowed the absorption of light in the visible region causing the efficient removal of sulfamethoxazole (SMX) in all tested conditions. The effect of different radiation sources for the CEC mixture degradation using noble metals doped onto TiO_2_ as a catalyst has also been addressed [57]. Despite the low bandgap of catalysts, visible light presents the lowest performance when compared to UV-A and sunlight radiation [67]. In fact, another important issue to evaluate the performance of visible light is the intensity of the different radiations considered. Moreover, it can be concluded that catalytic doping can promote stronger light absorption as the spectra are shifted in the visible region and consequently a higher rate of pollutants degradation. However, it is important to select a proper dopant to enhance the oxidation activity under visible radiation.

#### 3.1.3. Sunlight Radiation

Sunlight radiation is a clean and inexhaustible source of cheap energy that can be applied in the treatment of liquid effluents. In this view, the number of publications using solar radiation for photocatalytic detoxification and disinfection of water and wastewater is increasing [68,69]. As mentioned before, UV radiation has proven to be effective on photodegradation processes, namely for eliminating some pathogens, notably *Cryptosporidium*, which are resistant to disinfectants such as chlorine [70]. Although UV light represents 5% of sunlight, TiO_2_ performance under natural sunlight is very limited [71]. Therefore, TiO_2_ surface modifications are required to increase its efficiency in the absorption of natural sunlight. In this ambit, catalyst surface doping can be an interesting option [29]. Moreover, it also improves charge separation [72] avoiding fast recombination with a positive impact on the process efficiency.

To use sunlight radiation, a suitable reactor with a light collection system is often required. Abdel-Maksoud et al. [73] emphasized the geometry of compound parabolic collectors (CPC) as an effective accumulation system of UV radiation from sunlight, allowing the complete mineralization of Deoxytetracycline using TiO_2_. Several photoreactor projects for solar energy absorption have been tested for water and wastewater treatment [9,74].

Degradation efficiency is a powerful indicator to compare the performance of photocatalytic reactors of different types and geometries [74]. Some studies compared reactor configurations that use solar radiation as an energy source: cylindrical parabolic reactor; non-concentrated reactor and CPC [9]. Rodriguez et al. [75] used a photoreactor with a cylindrical parabolic concentrator for photocatalytic oxidation of Methyl Orange (MO) using TiO_2_ with activated carbon (AC). As a result, more than 90% removal has occurred, and the MO degradation with TiO_2_/AC using visible or sunlight was similar [75]. Non-concentrating photochemical systems are much more adapted to small-scale situations [76]. Composite parabolic cylindrical reactors have been seen as the best options for sunlight photocatalytic processes [76,77]. Although it has limitations related to the aging of glass tubes known as UV solarization, as well as the long exposure to solar radiation, further reduction of the UV transmittance of the tubes and implies additional costs for their regular replacement [78,79]. Several studies have shown that parabolic photoreactors can be successfully applied to remove very toxic compounds such as chlorophenols, pathogens, dyes, pesticides, chlorinated solvents, bacteria, and biorecalcitrant compounds in water [9,74].

Regarding the sunlight radiation, it is important to consider the time of the year and the place of the globe where the experiments were performed since the radiation flux and spectrum are different [79]. Gomes et al. [14] verified the impact of the season of the year on the sunlight radiation performance for the CEC abatement through photocatalysis and concluded that the presence of clouds and a low UV radiation index in November in Portugal can decrease significantly the photocatalytic oxidation performance on CEC removal. Moreover, this also can be related to the small fraction of UV-A and UV-B in the solar radiation spectrum during this time of the year [14].

### 3.2. Type of Catalysts

The selection of a suitable catalyst is a key point while designing a photocatalytic system. In the following sections, the types of materials tested in literature are discussed and their advantages and shortcomings are overviewed. In the photocatalytic processes for the wastewater treatment application, catalysts can be used as dispersed powder and coated or immobilized onto different supports.

#### 3.2.1. Powder Catalysts

Numerous materials, such as TiO_2_, ZnO, Fe_2_O_3_, WO_3_, CdS, ZrO_2_, MoS_2_, have been tested for photocatalytic applications [80]. CdS and Fe_2_O_3_ are semiconductors with unstable photocatalytic activity, in the whole pH range and undergo photocorrosion processes [81]. ZnO and TiO_2_ are UV light absorbers, often considered the most efficient semiconductors for promoting photocatalysis in aqueous solution [82,83].

TiO_2_ is non-flammable, poorly soluble, thermally stable, and non-hazardous metallic oxide [84]. ZnO is a non-toxic metal oxide that has a bandgap between 3.3 and 3.9 eV, slightly above TiO_2_ (3.2 eV) [85,86]. However, in the case of ZnO, photocorrosion often occurs with UV light, leading to a decrease in photocatalytic activity in aqueous solutions [87]. In this way, the usage of TiO_2_ as photocatalyst seems to be the most suitable option for these processes comparing the chemical properties. Furthermore, TiO_2_ has been reported as one of the most used semiconductors in the degradation of CEC [88,89]. In fact, the photocatalytic activity of TiO_2_ under UV light was considered the most effective for the hydroxyl radicals generation and applicable in a wide range of experimental conditions [90,91]. The photocatalytic reactions with powder TiO_2_ occur at the catalyst-substrate interface and thus it exhibits properties strongly dependent on the structure, surface, and morphology of nanocrystals [92]. A previous review emphasized that modifying TiO_2_ surfaces with metals and non-metals according to morphologies or scales allows an improvement in the adsorption capacity of contaminants, which is useful for water treatment [93]. These authors have noted that the use of TiO_2_ with a higher specific surface area led to better results than the use of larger quantities of semiconductors. Pereira et al. [72] evaluated the photocatalytic degradation of an aqueous solution containing 20 mg L^−1^ of the antibiotic Oxytetratracycline with TiO_2_ charges ranging from 0.1 to 0.5 g L^−1^ using simulated solar radiation, by an experiment carried out in a pilot plant equipped with CPC. The results proved that 0.5 g L^−1^ of TiO_2_ at pH 4.4 was the best combination to achieve 100% removal after 40 min of irradiation under 7.5 kJ L^−1^ UV dose. The photocatalytic degradation of the herbicide Metolachlor with TiO_2_ powder was studied and a degradation rate of 88% was achieved, despite the high toxicity of the byproducts generated [94]. Indeed, it has been reported that the superiority of photocatalytic activity of TiO_2_ (anatase) is attributed to differences in the Fermi level and the superficial hydroxylation extensions of the solid [95].

It must be noted that the degradation of contaminants in heterogeneous processes with TiO_2_, involves the mass transfer of pollutants from the liquid phase to the surface of the catalyst; Adsorption of reactants onto the surface (chemisorption or physisorption); Reactions at the adsorbed phase; Desorption of the product/intermediates from the surface of the catalyst and removal of the product from the interface to the solution [96]. Thus, the main limitations of using TiO_2_ powder are related to the possibility of sedimentation and the agglomerations formed in slurry reaction systems [97,98] which will reduce the catalyst availability and increase mass transfer resistances. Even so, a concentration of 0.5 g L^−1^ of TiO_2_ (P-25) under sunlight showed significant action in the inactivation of *E. coli* in water [99].

Regarding practical applications, the optimal load of catalyst must be chosen to avoid the excess of catalyst and guarantee full absorption of efficient photons. As an exampled, in laboratory experiments using a batch photoreactor, an optimal concentration of TiO_2_ of 2.5 g L^−1^ was found, while for the solar reactor CPC, which is a flow recirculation reactor corresponding to a batch reactor, only 0.2 g L^−1^ was considered as the optimal concentration [100]. The main limitations mentioned in the literature of using TiO_2_ powder are related to the inefficient exploitation of visible light, the low adsorption capacity of hydrophobic contaminants, and difficulties of separating the catalyst after reaction [101,102].

#### 3.2.2. Supported Catalysts

**Figure 2:**
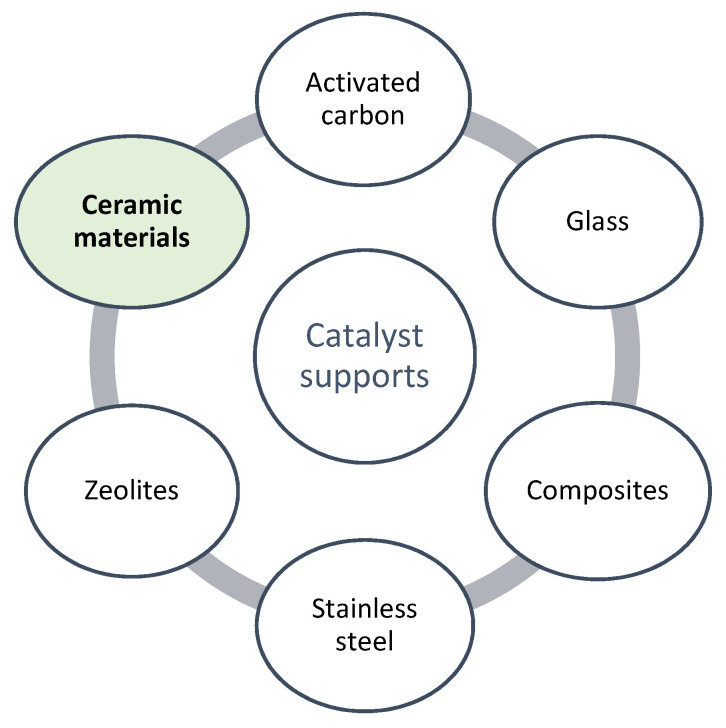
Different catalyst supports that can be used for TiO_2_ immobilization.

As aforementioned, the utilization of powders as catalysts (e.g., TiO_2_, P-25) has several drawbacks [103]. In particular, its separation from the reaction mixture is difficult, which makes it less reusable and increases the process capital and operating cost. To overcome this limitation, a variety of materials have been used as catalytic supports, to facilitate catalyst recovery from the reaction mixture [104,105]. It is widely known that with the use of immobilized catalysts, the surface area decreases significantly, and since most oxidation-reduction reactions of contaminants in an aqueous medium occur at the surface of the catalyst, the efficiency decreases as well. Therefore, the photoreactor configuration may also be an important point, and thus it is essential to favor the lighting of the catalyst [76]. In this context, as indicated in Figure 2, several supports were successfully tested to incorporate TiO_2_ in the removal of CEC, namely ceramic materials, activated carbon, glass, composites, stainless steel, zeolites, among others [38,83,104]. Among these, ceramic materials stand out because they are inexpensive, clays are highly available in the earth’s crust, porosity can be controlled, and are characterized by good thermal, chemical, and mechanical stability [105]. Anyway, different commercial photocatalysts (P25, P90, PC500, C-TiO_2_) were successfully immobilized in a composite material made of cement, clay, and wood fibers and demonstrated efficiency in the photocatalytic degradation of phenol [104].

From a practical point of view, it has been reported in several surveys that the ideal support for photocatalysis must satisfy several criteria as follows: (i) strong adhesion between catalyst and support; (ii) no degradation of catalyst reactivity by the bonding process; (iii) offer a high specific surface area; (iv) have a strong affinity for adsorption with pollutants [106,107,108]. Therefore, this review focused mainly on the different types of ceramic materials applied as support for the immobilization of TiO_2_.

## 4. Photocatalysts Supported in Ceramic Materials

Supported catalysts have gained increasing relevance for the degradation of pollutants [107,109]. Moreover, since TiO_2_ exposed to visible light is not very efficient, doping it with other substances may improve catalytic capacity [110,111]. However, it is important to note that the physical and chemical characteristics of ceramic supports can influence the photocatalytic activity of TiO_2_.

It is noted that the global demand for ceramic materials with comprehensive applications in the environment and other scientific areas is increasing [105,107].

In the further sections, a literature overview will be made regarding the most common ceramic materials used as TiO_2_ supports as well as the incorporation methods.

### 4.1. Ceramic Supports

Ceramic materials have proven to be excellent supports for TiO_2_ both in the degradation of air pollutants as well as in the removal of contaminants in aqueous solutions [112,113]. Indeed, ceramics have high-temperature resistance, extreme hardness, high chemical inertia, and lower density than metals [22,114]. There are two types of ceramic materials usually applied in this context, namely traditional and advanced ceramics. The former are made up of inorganic non-metallic solids (either nonmetallic or metallic compounds) and includes clay (plastic materials), silica (filler), and feldspar (fluxes) while the latter are made up of oxides, nitrate compounds, carbides, and non-silicate glass [105]. Regarding advanced ceramics, there is a growing interest in ceramic membranes based on Al_2_O_3_, TiO_2_, SiO_2_, and ZrO_2_ that demonstrate good performance in water treatment, turbidity removal, desalination, wastewater treatment, and other important industrial applications, apart from other advantages inherent in energy saving [115,116]. However, the preparation of nanocomposites using carbon, polymer, and ceramic materials is still at an early stage, requiring further investigation [117].

In this literature overview, the focus will be given to the traditional ceramics, since these materials present potential to be applied for the circular economy purpose. For example, clays are ubiquitous constituents of the Earth’s crust that serve as raw materials for traditional ceramics [118]. Clay minerals such as montmorillonite, bentonite, kunipia, kaolinite, smectite, rectorite, hectorite, laponite, palygorskite, halloysite, attapulgite, diatomite, and layered double hydroxides have been utilized as TiO_2_ supports [119]. Clay texture (1:1 and 2:1), optical properties, and surface area are factors that improve the performance of TiO_2_ photocatalytic activity. Thus, 2:1 clays (bentonite and kunipia-F) were considered better carriers of TiO_2_ than 1:1 clay (kaolinite) for photocatalytic degradation of MB and Chlorobenzene [119]. Experimental results of TiO_2_ composite on a photochemically stable clay for degrading herbicides (bromacil, alachlor, chlorotoluron, sulfosulfuron, imazaquin) and ammonia in aqueous solutions were significantly high, with removals ranging from 61 to 85% [38,120]. Moreover, Kaur et al. [82] supported doped and undoped TiO_2_ onto clay beads to promote degradation through UV light and solar radiation.

It should be emphasized that clay aggregates are of great interest due to the versatility of applications. In this context, the use of light expanded clay aggregate (Leca) arises interest due to its specific properties inherent to mechanical resistance, low density, and high porosity which means a higher surface area. Leca can enhance the mass transfer and allows proper contact between catalyst, light irradiation, and pollutant in an aqueous solution [121]. For these reasons, TiO_2_ supported in Leca can be an interesting solution for photocatalytic oxidation experiments. Zendehzaban et al., [38] comparing the performance of TiO_2_ and TiO_2_/(Leca + UV) photocatalysts and concluded that TiO_2_/(Leca + UV) composite improved its performance by up to 15%. Furthermore, there is no need to use a separation technology to recover the TiO_2_ from the liquid media [38]. Sohrabi and Akhlaghian [7] analyzed doped TiO_2_ supported in Leca (Cu/TiO_2_/Leca) activity for the phenol degradation under UV radiation. The CuO load enhances the incorporation of the dopant in the TiO_2_ mesh which increases the active sites available. In addition, the porosity of the Leca particles allows Cu/TiO_2_/Leca to be exposed to UV light in a better condition due to the higher surface area.

According to this, the porosity and roughness of support material can be relevant issues to establish good support for photocatalytic activity. Numerous works highlight the properties of ceramic materials as TiO_2_ support for photocatalytic degradation of organic pollutants [12,119]. Typically, the dispersion of TiO_2_ nanoparticles on clay mineral surfaces improves the photocatalytic activity of TiO_2_ due to high specific surfaces, high adsorption capacity, large pore volumes, chemical stability, and good mechanical properties [121]. It should be highlighted that the existence of photo-induced hydrophilicity on TiO_2_ coated porous substrates (e.g., clay tiles) is a medium that effectively prevents bacterial adhesion [122]. Meanwhile, the use of cetrimonium bromide as a surfactant of TiO_2_ solution for immobilization leads to a positive effect on the photoinduced surface hydrophilicity of the TiO_2_ films under UV light [123]. A new porous TiO_2_/fumed silica ceramic material using a 4% phosphoric acid binder was applied. The authors found that the transformation of TiO_2_ from anatase to rutile started at 725 °C. Moreover, the substrate exhibited good photocatalytic activity in the complete degradation of a 10 mg L^−1^ methyl orange solution using a radiation flux of 15 W.m^−2^ with ultraviolet light irradiation over 24 h [124]. Porous ceramics based on low temperature sintered diatomite were used as TiO_2_ support in the degradation of 5 mg L^−1^ of a Malachite Green solution, providing 86.2% removal after 6 h of ultraviolet irradiation [64]. For example, Szczepanik [121] highlighted that dispersion of TiO_2_ nanoparticles on clay mineral surfaces improves the photocatalytic activity of TiO_2_, providing more active surface sites and reducing the agglomeration of TiO_2_ particles in the reaction media. Carneiro et al. [112] developed photocatalytic ceramic materials by deposition of TiO_2_ nanoparticle layers and found that composites can act both to reduce air pollution and to decompose an aqueous MB solution. The efficiency was related to the porosity and roughness of the material. Recent studies on photocatalytic degradation of organic dye through the TiO_2_ layer deposited on single-flow ceramic membranes have revealed their photocatalytic efficiency. Convective flow through the pores of the TiO_2_-coated membrane has improved the mass transfer and, consequently, the photodegradation. The removal of the MB increased by up to 50% with an increase in the thickness of the coating (up to 6 nm) and the intensity of ultraviolet light (UV-A, 365 nm; 2 mW/cm^2^). This shows that a continuous photocatalysis process associated with membrane filtration has a significant potential for the treatment of polluted water [125,126]. Moreover, other parameters can be considered for the performance of the photocatalytic activity of different supports, such as the reaction pH, the catalyst dosage onto the support, the irradiation flux, the reactor geometry, etc. Pinato et al. [127] applied TiO_2_-coated alveolar clay foam as a photocatalyst achieving maximum removal of cumene hydroperoxide (CHP) in water using UV-A as a light source. The recyclability has been proven with 94–96% of CHP removal during four runs.

### 4.2. Other Alternative Supports

Alternative support materials can be used such as zeolites, glass spheres, and cement-based materials. Castañeda-Juárez et al. [128] supported doped and pure TiO_2_ on clinoptilolite to diclofenac degradation using solar radiation and evaluated the effect of several operating parameters on the oxidation efficiency. Miranda-Gárcia et al. [129] reported the incorporation of TiO_2_ in glass spheres for degradation of 15 CEC at low concentrations in simulated and real municipal wastewater. The experiments were carried out in a CPC reactor of the solar plant of the Almeria Solar Platform (Spain). The main results indicated 85% of the compounds were degraded within 120 min of lighting time, showing the potential of this technology as a good alternative to suspension systems for the treatment of polluted water.

Cement materials have limitations in incorporating TiO_2_ due to the reduction of specific surface area, low diffusion, and light transmission performance [27,130]. Zhao et al. [27] synthesized a novel lightweight aggregate-photocatalytic ceramsite sand, activated with the negative pressure method. This material proved to be an alternative for the application of photocatalysts (TiO_2_) in cementitious materials.

Besides glass beads, other materials such as silica beads have been referred to as excellent supports to the degradation of up to 100% organic compounds in liquid effluents [38,127,131]. Robust bonding between the supports and TiO_2_ is crucial for long-term application to avoid catalyst loss and water secondary contamination with TiO_2_ nanoparticles [91].

### 4.3. Immobilization Techniques of TiO_2_ onto Ceramic Materials

Several methods have been developed for the preparation of TiO_2_ nanocomposites immobilized in ceramic materials, namely sol-gel, dip-coating, impregnation method, chemical vapor deposition (CVD), electrophoretic deposition (EPD), and electrochemical treatment [38,127,131].

**Figure 3:**
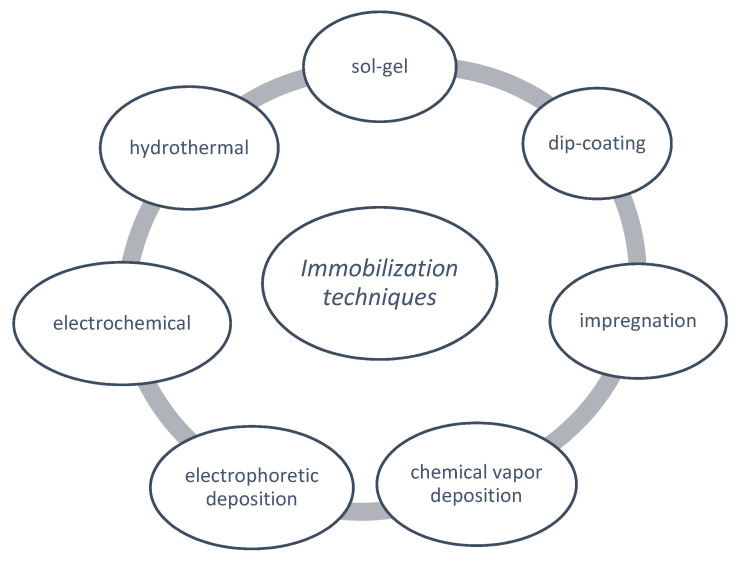
Different immobilization technologies used for TiO_2_ supporting.

This review will focus only on the most used methods for embedding ceramic substrates, namely the ones indicated in Figure 3.

The sol-gel method is a process for synthesizing materials from a liquid solution of organometallic precursors [132]. Titanium (IV) butoxide, titanium isopropoxide, and titanium tetrachloride (TiCl_4_), inorganic salts, are the precursors generally used for the synthesis of TiO_2_ nanocomposites using the sol-gel method [25,107]. Among the precursors in the sol-gel method, titanium isopropoxide can be solubilized in alcohol, and in this solution, two simultaneous reactions occur, namely hydrolysis and condensation. In this case, hydrolysis is the main chemical reaction that leads to the transformation of precursors into oxide monomers, and condensation is responsible for grouping these monomers to form a chain [25,133]. Belet et al. [134] compared two synthesis processes by the sol-gel method, aqueous synthesis, in which the reaction takes place in water, and organic synthesis, in which the reactions take place in an organic solvent and only a quantity of stoichiometric water is added to the reaction medium. The photocatalysts were coated on glass slides, noting that the aqueous and organic synthesis produced equally thick layers. However, the latter had better results in testing pharmaceutical degradation in water, while the former method had the advantage of being cheaper and consuming less energy. Moreover, the sol-gel method allows strong adhesion of the coating to the substrate and can be performed at ambient temperature [132]. Nevertheless, some disadvantages of the method concern the wide variation in particle size distribution and the need for a calcination step for crystallization, which can result in the melting of the substrate [25,107].

The dip-coating method allows the direct immobilization of the TiO_2_ powder and does not need precursors like the sol-gel method. This process is very similar to the impregnation method the difference consists in the form of contact between substrate and dispersion solution. The form of the produced substrate by this method is generally based on the use of suspensions, aqueous or alcoholic of TiO_2_ particles. The material adsorbed on the substrate undergoes a heat treatment to enable the particles to be calcined. Shan et al. [107] mentioned that dispersion coating and dip-coating are the two widely used methods for applying sol-gel to the substrate. It was found that the dispersion method is suitable for making a thick film, whereas dip-coating is applicable for producing a thin film on the overall surface of the support.

The impregnation is one of the most used methodologies for the immobilization of TiO_2_ over several supports [38,135]. Typically, the titanium dioxide is dispersed in the alcohol solution to produce the slurry and then this suspension is placed onto the support left to stand for some time until the solvent evaporation whereas the dip-coating has short periods of contact [135]. In the case of alcoholic suspensions, the procedure consists of preparing a suspension with ethyl alcohol and with an acidity suitable for the adsorption of TiO_2_, which after filtration and drying at 100 °C must be calcined at 550 °C for 30 min [38,135]. In this case, the substrates incorporated in the suspension will be calcined with enough volume of TiO_2_ adsorbed. Alternatively, in the impregnation method, the solvent evaporation stage can be made with microwave assistance, since can result in a more efficient junction between TiO_2_ and support [136].

The chemical vapor deposition (CVD) method is based on the deposition of thin solid oxide films on a heated substrate using vapor phase mixtures of metal-containing precursors (in the case of oxide deposition these are usually metal alkoxides or β-diketonates) and an oxidant [137]. CVD may use different types of precursors and may involve atmospheric deposition under chemical vapor pressure [107]. In a typical CVD method for the formation of a titanium oxide film, a vapor of titanium tetrachloride (TiCl_4_) is hydrolyzed by reaction with water vapor at the surface of a substrate to form a film of a titanium oxide precursor. The substrate is then calcined to convert the titanium oxide precursor into titanium oxide. TiCl_4_ used as a reactant may be replaced by another hydrolyzable titanium compound such as a titanium alkoxide, but from a commercial standpoint, the use of TiCl_4_ is advantageous since it is less expensive and has a lower boiling point [138]. A steam deposition apparatus suitable for use in the film-forming process is required. According to [25] CVD synthesis method falls between two categories either, chemical or physical process.

The method of electrophoretic deposition of TiO_2_ powder in the form of a film on a metallic substrate has been referenced and with results that demonstrate adhesion of TiO_2_ in stainless steel, and when used in photocatalytic tests of water purification, no titanium release has been observed [139,140]. The working principle of EPD is based on the movement of charged particles in an electric field. The particles move towards the working electrode due to the applied cell voltage and their accumulation on this electrode leads to the formation of a homogeneous layer [107,139,140]. This process has several advantages since it is cost-effective and also possible to coat complex-shaped substrates, besides simple planar substrates [140].

The electrochemical method requires an electrolyte solution to promote electroconductivity between the sacrificial organic and sacrificial electrodes. In this method, TiO_2_ is dispersed in an electrochemical cell that contains the support, solvent, and sacrificial organic substance. This cell is placed in an electrolyte solution as well as the other electrochemical cell containing the sacrificial electrodes [128]. The application of this method for catalyst supporting must consider the current density and treatment time as important parameters [128]. Castañeda-Juárez et al. [128] used an electrochemical treatment method to support clinoptilolite zeolite with TiO_2_ doped with Cu, Fe, and Fe/Cu, with relevant efficiency on the diclofenac degradation through photocatalysis. In this work, the sacrificial organic and solvents were water and ethanol, and the sacrificial electrodes were Fe, Cu, or both.

The hydrothermal method consists on the application of pressure and temperature to promote the adhesion of TiO_2_ onto the support [120,135]. Paul et al. [120] analyzed different temperatures, 100, 150, and 200 °C during 24 h to support TiO_2_ onto Laponite for the photocatalytic oxidation of herbicides. According to the results, the high temperature of hydrothermal treatment promotes the better photoactivity of supported TiO_2_ [120]. This method for TiO_2_ anatase immobilization can promote the appearance of the rutile phase or increase their concentration with the duration of hydrothermal treatment [135]. Typically, this method can be applied in autoclaves [120] but recently some authors use microwave assistance to promote the hydrothermal treatment [135].

Regardless of the immobilization technology selected, almost all of them comprise the calcination step as a final stage. This step will promote the immobilization of TiO_2_ onto the support surface.

Thus, each immobilization technique has advantages and disadvantages, while the preparation procedures and the substrates used directly influence the photocatalytic activity of TiO_2_ [107]. In this way, during the preparation of the catalysts is important to select the most suitable technology according to the ceramic support used and the final application. The immobilization of TiO_2_ in ceramics by different methods has been considered a sustainable alternative in wastewater treatment [40,135,141]. Table 1 summarizes studies from the literature related to ceramic supports and the incorporation methods applied in the treatment of liquid effluents.

### 4.4. Catalyst Characterization

After a photocatalyst immobilization onto the support, several characterization techniques can be used to analyze the morphology and the homogeneity in the distribution of TiO_2_ within the support. In fact, the dispersion of TiO_2_ onto the support is important for the effectiveness of the catalyst in photocatalytic oxidation since the TiO_2_ can agglomerate, which can lead to an efficiency reduction [98]. In particular, biotemplates, including animal-based and herbal-based templates, can assist in TiO_2_ coating quality control and reduces the TiO_2_ crystallite size to 18 nm [146]. The most common techniques used to characterize these materials comprise the scanning electron microscope (SEM), X-ray diffraction (XRD), Fourier transform infrared spectroscopy (FTIR), and transmission electron microscopy (TEM).

The SEM technique makes it possible to analyze the morphology of the composite surface allowing the visualization of the dispersion of TiO_2_ onto the support. Moreover, Zendehzaban et al. [38] analyzed the SEM images for Leca before and after TiO_2_ immobilization and concluded that the high porosity of Leca does not suffer significant modification after immobilization. The XRD can be useful to characterize the crystalline structure of materials as well as TiO_2_ polymorphs supported in ceramic materials [27,119]. Saleiro et al. [147] evaluated by XRD the TiO_2_ supported in structural ceramics and observed that the most photoactive phase (anatase) changed to the less photoactive phase (rutile) with the increase in sintering temperature. However, concluded that red ceramics tend to inhibit this transformation, concluding that at 700 °C the anatase phase is the major crystalline phase (about 50%). Zendehzaban et al. [38] verified that the TiO_2_ immobilization onto Leca does not promote considerable changes in the typical XRD patterns of TiO_2_ alone.

The FTIR allows assessing information about the structure of compounds, showing the degree of heterogeneous bonding within the particles [123,148,149]. Moreover, this technique provides information about the bonds between titanium dioxide and support. Through the FTIR results, it was revealed that the crystallinity and surface area of anatase nanocrystals have a more significant impact on photocatalytic activity [12,149]. This is because Ti-O-Si bonds are formed which stabilize the anatase nanocrystals and prevent loss of surface area. Moreover, FTIR techniques can provide information on the presence of any other impurities.

In addition, transmission electron microscopy (TEM) is a powerful technique to obtain very detailed micrographs [123]. For example, the presence of small openings formed by anatase crystals and delamination of Laponite (clay mineral) can be observed, as well as thermal shock and shrinkage in the framework during hydrothermal treatment and subsequent calcination result in the irregular ordering of the porosity. Meanwhile, Peikertova et al. [150] used Raman spectroscopy to study the effect of temperature on the stability of the TiO_2_ phases.

## 5. Photocatalysis with Supported Material

The application of supported TiO_2_ instead of powders aims to facilitate the catalyst reuse and recovery. The efforts to improve efficiency in the application of this technology has been stressed by Yang et al. [151] regarding:

(i) increasing light-harvesting capacity via defect engineering.
(ii) enhancing charge separation via interface engineering.
(iii) accelerating surface reaction.

In the following sections, the application of supported TiO_2_ photocatalysts for pollutants removal from water is overviewed.

### 5.1. Chemical Contaminants of Emerging Concern Removal

The efficiency of the treatment process is in general analyzed based on the removal capacity of the target contaminants [152]. Meanwhile, the optimization of the operating conditions such as pH, catalyst load and temperature, can improve the removal of chemical compounds from liquid effluents [38,127].

The studies involving the degradation process of sulfamethoxazole (SMX) with TiO_2_ supported on expanded perlite, show that pH did not influence the adsorption tendency of SMX. On the contrary, the highest photodegradation occurs in the less adsorbed anionic form. This suggests that the pH-induced changes in reactivity may be related to the changes in the SMX molecule and not in the surface of TiO_2_ [103]. Other studies [153] demonstrated that immobilization of TiO_2_ in glass plates together with UV radiation has been effective in removing ciprofloxacin (CIP) in aqueous solutions. The removal efficiency of CIP in synthetic and real effluent was 92.8% and 86.6%, respectively. In addition, the removal of the antibiotic had a direct relation with contact time and an inverse relation to the initial concentration of CIP and pH (3 and 5). Concerning adsorption contribution, [154] showed that the titania–clay heterostructures have significantly more affinity for Rhodamine B (RhB) than for phenol, and the photocatalysts prepared show promising activities in the degradation of RhB and phenol in water using solar light. In the case of phenol photodegradation, after 24 h of irradiation, the mineralization reaches very high values, with almost complete degradation of phenol and aromatic compounds and a decrease in the value of the initial ecotoxicity.

The photocatalytic activity of TiO_2_ in ceramic substrates has been quantified with respect to several contaminants, and Table 2 summarizes the performance achieved in liquid effluents. The most common methods for preparing supported photocatalysts are based on thermal treatment and the sol-gel technique. Removals ranged from 60 to 100% revealing that the incorporation of TiO_2_ in ceramic substrates has been efficient and, in some cases, superior to powder P-25 [28,143].

The mesoporous structure of clay mineral particles allows exposure to light of the crystalline anatase particles and the diffusion of organic molecules to their surfaces [119,120]. Some authors [120] emphasized that the porosity and crystalline size of TiO_2_ as important parameters for catalytic activity and adsorption of pollutants to the active sites of the catalyst. Indeed, the performance of heterogeneous photocatalysis involves among other parameters the size of the anatase crystals, porosity, and pH [119,155]. Highly crystallized and high porosity anatase samples allow the access of large organic molecules as well as light during the reaction [120]. pH can influence differently the degradation of contaminants [38,156] because, in acidic or alkaline conditions, the TiO_2_ surface can be protonated or deprotonated [156,157].

The photocatalytic decomposition rate of CEC in liquid effluents using TiO_2_ supported on ceramic materials under UV or visible light decreases gradually over time according to the Langmuir-Hinshelwood model, according to Equation (10) [38,103].

(10) $$r_{R}=\frac{-dC_{R}}{dt}=\frac{K_{r}KC_{R}}{1+KC_{R}}$$

where *r_R_* is the degradation rate, *C_R_* is the concentration, *t* is the reaction time, K_r_ and K are reaction and adsorption constants, respectively.

**Table 2:** Contaminants removal with immobilized TiO_2_ on ceramic substrates application.

Reference	Support	Incorporation Method	Pollutant	Operating Conditions	Efficiency (%)
[7]	Leca (doped with Cu)	Sol-gel	200 ppm of phenol	UV light, catalyst amount (0.5 g L^−1^); aerated	61 (2 h)
[27]	Lightweight aggregate-photocatalytic ceramsite sand	Vacuum ultrasonic method	2 µL of benzene	UV light, catalyst amount (4 g L^−1^)	100 (3.3 h)
[38]	Leca	Impregnation method	0.05 M of ammonia	UV light, catalyst amount (0.5 g), 750 mL of solution, pH 11	85 (5 h)
[120]	Laponite	Hydrothermal method	10 ppm of Bromacil and Alachlor; 5 ppm of Chlorotoluron, Sulfosulfuron, and Imazaquin	UV light as a radiation source, Catalyst amount 1 g L^−1^ of solution	60 to 100 (1 h)
[135]	Perlite	Dip-coating	1 mM of phenol	UV light, 11 g L^−1^ catalyst amount; aerated	83.3 (4 h)
[141]	Porous ceramic	Sol-gel	5 mg L^−1^ of Rhodamine B	UV light, catalyst charge 140 mg L^−1^	83 (2 h)
[156]	Reticulated Al_2_O_3_ ceramics	Dip-coating	20 mg L^−1^ of RO16 azo dye (Dystar);	UV-C, catalyst amount (20 g); 500 mL dye solution	≈ 100 (1.25 h)
[157]	Sepiolite (Sep)	Sol-gel	2.0 × 10^−5^ mol L^−1^ of eosin dye	UV light, 1.0 g L^−1^ of the catalyst, neutral pH	72 (2.5 h)
[158]	Ceramic material	Sol-gel	5 mg L^−1^ of MB	UV-C, catalyst amount (0.4 g) inject air into the reactor	60 to 70 (1 h)
[159]	Clay beads	Dip-coating	25 mg L^−1^ of pesticide Monocrotophos	UV light, catalyst amount (26.8 g), 2 L of solution, pH 5	78 (7 h)

### 5.2. Disinfection Capacity

Disinfection using TiO_2_ under light effect has been investigated due to the potential for destruction of membrane cells by the hydroxyl radicals [131]. The prevention of regrowth and inactivation of microorganisms with lower sensitivity to solar irradiation are among the main advantages of introducing photocatalytic materials for solar water disinfection [26]. Inactivation rates of viruses and bacteria were relatively high in photocatalytic processes with supported TiO_2_ [160,161]. In a previous review [162] the effect of photoactivated TiO_2_ on microorganisms was demonstrated. This technology can deactivate and even kill a wide range of Gram-negative and Gram-positive bacteria, filamentous and unicellular fungi, algae, protozoa, mammalian viruses, and bacteriophage. Virus inactivation kinetics have been associated with an improved process of TiO_2_ adsorption [160,163]. Also, the doping of Pt/TiO_2_ and its subsequent coating on ceramic tiles have proven effective in removing chemical and bacterial pollutants from water [164]. Thus, Pt/TiO_2_/support ceramics can be applied in swimming pools, hospitals, water parks, and even industries for water decontamination. Just as TiO_2_/vermiculite composites showed the unique characteristic of floating on the water surface, where optimal illumination and oxygenation occurs, leading to a strong increase in their photocatalytic efficiency [165].

Despite few studies involving TiO_2_ disinfection capacity in ceramic substrates, its incorporation into the porous ceramic disk filter and Montmorillonite revealed optimal interaction with *E. coli* and *Staphylococcus aureus*, promoting a significant sterilization rate [166,167]. Specific studies [168] compared the effect of TiO_2_ and TiO_2_ particles coated in a ceramic plate on the surface antigen of the Hepatitis B virus (HBsAg). The results showed that with TiO_2_ suspension 97% of HBsAg (pH 7.2) was destroyed, while with the ceramic plate as support about 94% of disinfection capacity was observed. In both cases, the destruction occurred under UV irradiation (365 nm) for 4 h. Probably suspensions of TiO_2_ have a higher impact on disinfection in comparison to immobilized catalysts [99,169]. However, suspension-based photocatalysts are very difficult to separate from treated water after usage. Some studies [170] concluded that a high load of TiO_2_ can generate a scavenger effect, which in turn leads to a reduction in the efficiency of the catalyst in inactivating bacteria.

### 5.3. Toxicity Assessment

The performance of effluent treatment technologies should also be assessed in terms of their impact on water toxicity [50,171].In fact, the by-products formed during the AOP treatment may be more toxic than their precursors [26,172]. Therefore, toxicity tests are essential for wastewater treated with AOP before disposal.

Xing et al. [173] evaluated the toxicological data of the CIP degradation process by N-TiO_2_ immobilized in glass beads. The consensus method was used to evaluate the CIP toxicological data, including LC_50_ (Average Lethal Concentration in 96 h). The results showed that most of the 17 degradation products detected had lower toxicity than CIP, but some of the degradation products were more toxic.

Other studies [174] evaluated the chronic ecotoxicity of three pharmaceutical products, Metronidazole, Atenolol, and Chlorpromazine, before and after photocatalysis by immobilization of a TiO_2_ mixture namely PC-500 in ceramic plates by the sol-gel method. The results of the TOC (90% removal after 16 h) and ecotoxicological experiments with *Spirodela polyrrhiza* showed that UV/TiO_2_ could mineralize and effectively reduce the ecotoxicity of pharmaceutical products. Similar results were obtained by Alfred et al. [175] on the degradation of two antibiotics (Ampicillin and Sulfamethoxazole) and an antimalarial drug (Artemether) in water by incorporation of TiO_2_ on natural kaolinite clay, Na_2_WO_4_, and biomass. The photodegradation of these compounds was higher than 90% under sunlight. In addition, the concentrations of by-products of the mineralization process after photo-degradation are safely below WHO standard limits for drinking water.

The use of organisms for testing (bacteria, bivalves, fish, and algae), among others, can serve as a model indicator of the total toxic effect [14,172]. In fact, besides the referred information some of the ceramic materials used during the photocatalytic oxidation experiments can suffer leaching which, for example, can increase the presence of metals in water. This will create secondary contamination that can be detected by the aquatic species previously mentioned.

### 5.4. Water Recovery

To the best of our knowledge, the literature does not contain specific information on the reuse of treated wastewater by TiO_2_ supported by ceramic materials. Although heterogeneous photocatalysis has been studied for the removal of pollutants in aqueous matrices for two decades, its use in wastewater treatment is still at a stage of technological research [176]. However, several references are indicating that AOP with hydroxyl radical generation applying oxidants such as H_2_O_2_ and aided by TiO_2_ catalysts have promoted the reduction of cytotoxicity and mutagenicity within the permitted limits, ensuring that treated wastewater could eventually be suitable for industrial reuse, irrigation, and aquaculture [177,178]. For example, the efficient use of ZnO and TiO_2_ in the treatment of textile wastewater, revealed the potential to remove color and other physical and biological properties that guarantee the quality for its re-use for agricultural and domestic purposes [179]. Water reuse and recycling are the first of the five thematic priorities of the European Partnership for Innovation (EIP) that supports the advancement of water innovation solutions to 2020 [180]. In this scope, by applying 200 mg L^−1^ of TiO_2_ in a CPC-type pilot solar plant to treat effluent from a municipal wastewater treatment plant contaminated with 22 pharmaceutical compounds at moderate concentrations (maximum 680 ng L^−1^, except Diclofenac ~ *24* µg L^−1^ and Hydrochlorothiazide ~ 24 µg L^−1^) good efficiencies were found [181]. Moreover, the acute toxicity of the treated effluent was assessed by the percentage of inhibition of *V. fischeri* bioluminescence after 15 and 30 min. The results showed that the inhibition percentages were already low (<15%) from the beginning of the experiment, thus indicating that the effluent was not significantly toxic. Also, the efficiency of a CPC reactor [182] with TiO_2_ coated on glass beads for the treatment of wastewater with pesticide mixtures (Thiabendazole, Imazalil, and Acetamiprid), 100 μg L^−1^ each was evaluated. It was found that the degradation rate of Imazalil during the photocatalytic experiments was very high and was not affected by the presence of the other contaminants. Preliminary results show that the composite in question proved to be photocatalytically active and mechanically stable during the experiments. Therefore, the optimization and application for tertiary effluent treatment in citrus processing industries are recommended. Moreover, Sousa et al. [181] studied the TiO_2_ assisted photolytic and photocatalytic oxidation of the anxiolytic drug Lorazepam under artificial UV light and natural sunlight. They compared the photolytic and photocatalytic degradation kinetics of Lorazepam using two experimental systems: a laboratory-scale photochemical reactor supplied with a medium pressure UV mercury lamp (LsAUVP) and a solar pilot plant with compound parabolic collectors (SPP-CPC). Lorazepam was tested in its most marketed dosage form in Portugal, 1 mg (Wyeth). Preliminary results showed that using the LsAUVP apparatus, the highest degradation performance of Lorazepam was obtained by photolysis, while using the SPP-CPC system, the best degradation performance was obtained by photocatalysis with 200 mg L^−1^ of TiO_2_. Other efficient wastewater treatment alternatives involve combining two or more physico-chemical processes to maximize the removal of recalcitrant organic compounds [178,183].

The great interest in researching approaches to process optimization and integration to solve ecological risks and enhance removal efficiency should be highlighted. Thus, the recovery and reuse of wastewater by AOP in particular with TiO_2_ supported by ceramic materials can be a future contribution to conserving valuable water sources and thus mitigate the effects of climate change.

The reuse of recovered water in practice is lagging behind the political ambition. Concern about its impact on human and environmental health has been identified as a key barrier to its full recovery [184,185]. Among other aspects, the economic issue has been seen as attractive for reuse since it does not involve other investments with transport.

Although effluents in some cases have presented toxic intermediates, numerous publications show compatible levels of toxicity for the discharge of treated effluents as well as their reuse [172,183].

Yaqoob et al. [117] evaluated the effect of 100 mg L^−1^, 50 mg L^−1^, and 25 mg L^−1^ of TiO_2_ suspensions in wastewater treatment. The adverse effects of treated wastewater on maize growth attributes were significantly improved with a load of 25 mg L^−1^ (*p* < 0.05), whereas 100 mg L^−1^ load of TiO_2_ significantly inhibited seed germination, seedling growth and caused the accumulation of phenolic in maize plants (*p* < 0.05). In other studies [186] by using 0.5 g L^−1^ TiO_2_ under UV-A, in acid conditions (pH = 3) completely discoloring textile effluents was observed and a reduction of the chemical oxygen demand (COD) between 40% and 90% after 4 h treatment. A comparison of the photocatalyst (TiO_2_) synthesized by the sol-gel technique and coated on different substrates (transparent glass, glazed ceramic tile, and stainless steel) was carried out [187]. The coated substrates were used to remove the color from dyeing wastewater under UV irradiation (36 W-UV-A or 30 W-UV-C lamps) and pH 3–11. The results showed that the optimal substrate that produced the highest color removal efficiency (93.03 ± 0.66%) was TiO_2_ coated glass under UV-C irradiation. Some studies have demonstrated good results regarding adhesion and duration of TiO_2_ coating on ceramic materials in several test cycles, thus with positive perspectives to implement this approach on a larger scale for wastewater treatments containing CEC [131]. In this perspective, in the future, the technique may become competitive to boost the reuse of water for different purposes due to the low cost it presents.

## 6. Conclusions and Future Perspectives

The application of TiO_2_ as a slurry catalyst for the degradation of CEC in liquid effluents has some disadvantages due to the tendency of sedimentation with a decrease in the amount of light absorbed by the catalyst, and difficulties in avoiding the loss of catalyst that requires sophisticated and expensive methods of separation. In view of this, ceramic materials reveal excellent characteristics and properties as substrates for the incorporation of TiO_2_, exhibiting high levels of removal both in the degradation of air pollutants and contaminants in aqueous solutions. This is associated in part with the efficiency of light absorption as well as the porosity of ceramic substrates which effectively influence the intrinsic properties of TiO_2_, inhibiting energy dissipation and increasing the active sites of the catalyst. Several incorporation methods improve TiO_2_ adhesion to the substrate providing composite stability (TiO_2_ + substrate).

In this perspective, in the future, this treatment technique may become competitive to boost the reuse of water for different purposes due to its low cost and contribute to the conservation of valuable water sources and mitigate the effects of climate change. In this literature overview, it was possible to conclude that there is a knowledge gap related to the associated costs to immobilize nanoparticles onto ceramic materials. In fact, this can be one of the factors that need to be considered in such an approach. It is important to highlight that the cost associated with this technology can be decreased with the number of cycles of reuse without loose relevant efficiency. If the supported catalyst can be reused many times, the immobilization costs can be insignificant comparing with the operational costs powder nanoparticles catalyst recovering from the reaction medium. Moreover, the usage of supported ceramic materials over different cycles is relevant from the circular economy perspective due to the reduction of waste that will be produced in this case.

In relation to the recognized potential risks associated with the presence of emerging contaminants in the environment, assessments of the ecotoxicity of treated effluents serve as an indicator for monitoring the quality of wastewater for re-use purposes. Several aspects must be solved in the application of the technology with emphasis on assessments and reduction of ecotoxicity of treated effluents, energy dissipation, reactor configuration, increase of the surface area of the catalyst.

The appropriate load of the catalyst that prevents its agglomeration, concentration of contaminants, and pH should be optimized on a laboratory scale to increase the photocatalytic process on an industrial scale. Overall, it is noticeable that from an energy cost point of view, the technology presents advantages for the equatorial regions due to the higher incidence of sunlight per year.

In fact, future industrial wastewater treatment likely encompasses the usage of immobilized TiO_2_ through the sunlight radiation. Regarding this, the main challenge is to find a suitable ceramic substrate, since this kind of material can have reliable properties as support, even from a circular economy perspective. Indeed, ceramic materials can be considered for other applications at the end of their life cycle. Moreover, the reduction of the bandgap to make TiO_2_ more active under visible sunlight radiation is another relevant feature for the photocatalytic oxidation performance. This reduction of bandgap can be achieved with metallic or non-metallic species. In this way, one of the best solutions for wastewater treatment is the immobilization of doped TiO_2_ onto suitable ceramic support to be used in reactors with higher solar exposure areas. This solution may have a higher lifetime without a significant loss of efficiency.

## Figures and Tables

**Table 1:** Ceramic supports and their incorporation methods.

Ceramic Support	Incorporation Method	Application	Reference
Leca	Deposition of TiO_2_ solution in Leca followed by calcination at 550 °C	Photocatalytic degradation of ammonia	[38]
Diatomite-based porous ceramics	Hydrolysis deposition method with TiCl_4_ as the precursor	Degradation of malachite green solution	[64]
Ceramic materials	Dip-coating by immersing ceramics for 30 min followed by drying at 85 °C for 30 min and then 150 °C for 5 min	Self-cleaning properties to ceramic surfaces	[112]
Clay nanocomposites	Hydrothermal synthesis (T = 100 °C and P = 5 atm) during 24 h	Advanced treatment of wastewater loaded with dye (MB) and heavy metal (cadmium)	[119]
Laponite	Hydrothermal method (T = 100, 150 and 200 °C)	Photocatalytic degradation of Herbicides	[120]
Ceramic membranes	Atomic layer deposition of TiO_2_ onto anodized aluminum oxide membranes	Photocatalytic degradation of MB	[126]
Porous ceramic material	Sol-gel method, titanium tetra-isopropoxide was used as TiO_2_ precursor	Degradation of Rhodamine B 2020	[141]
Clay roofing tiles	Spray method	Inactivation of *Pseudomonas* *Aeruginosa* bacteria and degradation of *p*-chlorobenzoic acid the mesoporous coating	[142]
Glazed ceramic pieces	Spraying with P-25 followed by calcination at 450 °C	Degradation of methylene blue (MB)	[143]
Ceramic tiles	Sol-gel coating for SiO_2_/TiO_2_ preparation followed by deposition technique on ceramic tiles	Degradation of MB	[144]
Ceramic substrate (Ca_2_MgSi_2_O_7_: Ce^3+^ and kaolin)	TiO_2_ (P25, Macklin) powder was dispersed in 5 mL deionized water by sonication. Then the dispersion was dropped onto the surface of the Ca_2_MgSi_2_O_7_: Ce^3+^ ceramic substrate followed by calcination at 400 °C	Water purification	[145]
